# Effect of acupuncture on *Deqi* traits and pain intensity in primary dysmenorrhea: analysis of data from a larger randomized controlled trial

**DOI:** 10.1186/1472-6882-14-69

**Published:** 2014-02-21

**Authors:** Guang-Xia Shi, Qian-Qian Li, Cun-Zhi Liu, Jiang Zhu, Lin-Peng Wang, Jing Wang, Li-Li Han, Li-Ping Guan, Meng-Meng Wu

**Affiliations:** 1 Acupuncture and Moxibustion Department, Beijing Hospital of Traditional Chinese Medicine, affiliated to Capital Medical University, 23 Meishuguanhou Street, Dongcheng District, Beijing 100010, China; 2 School of Acupuncture-Moxibustion and Tuina, Beijing University of Chinese Medicine, 11 Bei San Huan East Road, Chao Yang District, Beijing 100010, China; 3 The First Hospital affiliated to Tianjin University of Traditional Chinese Medicine, 312 West Anshan Avenue, Tianjin 300193, China

**Keywords:** Acupuncture, Deqi traits, Pain intensity, Primary dysmenorrhea

## Abstract

**Background:**

*Deqi* is a central concept in traditional Chinese acupuncture. We performed a secondary analysis on data from a larger randomized controlled trial (RCT) in order to assess the effect of acupuncture on *deqi* traits and pain intensity in primary dysmenorrhea.

**Methods:**

A total of 60 primary dysmenorrhea patients were enrolled and randomly assigned to one of three treatment groups. Acupuncture was given at SP6, GB39 or nonacupoint. Subjective pain was measured by a 100-mm visual analogue scale (VAS) before and after acupuncture. The Massachusetts General Hospital acupuncture sensation scales (MASS) with minor modification was used to rate *deqi sensations* during acupuncture.

**Results:**

The results showed that VAS scores of pain after acupuncture were significantly decreased comparing to before acupuncture treatment in all three groups (*P* = 0.000). However, no significant differences were found among three groups at the beginning or end of acupuncture treatment (*P* = 0.928, *P* = 0.419).

**Conclusions:**

There was no statistical difference among three groups in terms of intensity of *deqi* feeling. The types of sensation were similar across the groups with only minor differences among them.

**Trial registration:**

Trial registration number: Controlled-Trials.com ISRCTN24863192.

## Background

Acupuncture has been increasingly used as an alternative and complementary therapy in various clinical conditions, especially in the pain management [1-3]. But its mechanism is still unclear. According to both ancient traditional Chinese and modern text books, the *deqi* feeling of patients is different, which is referred as *suan* (aching or soreness), *ma* (numbness or tingling), *zhang* (fullness/distention or pressure) or *zhong* (heaviness) around the acupuncture point and/or along the meridians [4,5].

Recent controversy in the field of acupuncture research has been generated when several large scale RCTs showed no significant differences between acupuncture and minimal or sham acupuncture. But compared to control cases, the clinical effects of acupuncture treatment are significantly positive [6-8]. It claims that *deqi* is important in creating a positive clinical outcome [5,9]. Non-penetrative “placebo needles” such as the streitberger needle also elicited *deqi*, which presenting a problem when acupuncture is evaluated within controlled trials [10,11]. Thus, *deqi* may have implications both for clinical practice and trial design. *Deqi* traits mean the nature of the sensation and intensity. There is no consensus for a method or instrument to quantify *deqi sensations*. Particularly, few studies have investigated the effect of acupuncture on different aspects of *deqi* or pain relief [12,13]. We had previously designed AAEPD-II to investigate immediate effects of acupuncture at a specific acupoint compared with unrelated acupoint and nonacupoint among primary dysmenorrhea patients. The present data was a secondary analysis from the main RCT to assess the effect of acupuncture on different aspects of *deqi* and pain relief in primary dysmenorrhea.

## Methods

### Study design overview

The main study was designed as a multicentre RCT with a sample size of 501 in six large hospitals of China (i.e., Dongzhimen Hospital affiliated to Beijing University of Chinese Medicine, Beijing Hospital of Traditional Chinese Medicine affiliated to Capital Medical University, Huguosi Hospital of Traditional Chinese Medicine affiliated to Beijing University of Chinese Medicine, China-Japan Friendship Hospital, the First Hospital affiliated to Tianjin University of Traditional Chinese Medicine, and the Hospital affiliated to Shandong University of Traditional Chinese Medicine). We used the subset of the data for this paper only from one hospital (the First Hospital affiliated to Tianjin University of Traditional Chinese Medicine) with a total of 60 primary dysmenorrhea patients. That is, 23 patients in SP6 acupuncture group, 26 in GB39 control group and 11 in Nonacupoint control group, respectively. The methodology is the same with the main study [14].

The trial protocol has been approved by the Research Ethical Committee of the First Hospital affiliated to Tianjin University of Traditional Chinese Medicine, and the study itself was conducted according to common standard guidelines (Declaration of Helsinki, Good Epidemiological Practice: http://www.dundee.ac.uk/iea/GoodPract.htm). Written informed consent were obtained from participants or caregivers if children under the age of 18.

### Outcomes

Demographic measures collected at the baseline evaluation included age, menstrual cycle and baseline pain intensity.

#### Pain intensity

This was measured on a standard of 100-mm VAS (0 = no pain, 100 = worst pain ever), before and after acupuncture. Mean pain scores were calculated for both pre- and post-acupuncture dysmenorrhea pain.

#### *Deqi* sensations

*Deqi* was monitored with the MASS [12]. Seven most common descriptors in Chinese, which were soreness, numbness, heaviness, warmth, cold, sharp pain and dull pain, were selected for minor modifications. Immediately after the needle was inserted into the skin, the subject was asked by another researcher if *deqi sensations* occurred during the stimulation. And the intensity was rated on the scale of 1–100 mm.

The “Acupuncture Sensation Spreading Scale” was also used to rate the localization and expansiveness of *deqi sensations* along the lower limbs [13]. Orienting marks on this separate continuum include “none”, “localized”, “ankle”, “upper legs”, “lower legs”, and “knee-joint”, ranging from 0 to 5 scores. Others surrounded kept quiet during the whole intervention until the questionnaire was assessed and filled in.

### Statistical analysis

The SPSS 13.0 for Windows statistical software was used in the statistical analysis. Mean ± Standard Error (Mean ± SE) was given for each parameter. One-way ANOVA or rank sum test were used to compare the between-group differences for quantitative data. Enumeration data were presented as frequency using chi-square test or Fisher exact probability test. The significance was set at *P* < 0.05.

## Results

### Demographic data of subjects

There were no significant differences at the baseline among three groups. On average, participants were at 22.4 ± 2.8 years of age and had experienced pain for about 83.2 ± 40.7 months. The average baseline score on the VAS was 64.8 ± 16.8 mm for pain intensity of dysmenorrhea.

### Comparisons of pain intensity

Comparisons of the pain intensity before and immediately after acupuncture were presented in Table 1. Compared to pre-acupuncture, VAS scores in three groups were significantly decreased after acupuncture treatment (*P* = 0.000). However, the VAS scores showed no significant differences among three groups at the beginning of acupuncture (Pre-acu, *P* = 0.928). Meanwhile, no significant differences were detected among patients in three acupuncture groups after intervention (Post-acu, *P* = 0.419).

**Table 1:** VAS scores of pain intensity pre- and post-acupuncture (mm, Mean ± SE)

**Group**	**N**	**Pre-acu**	**Post-acu**	*P
SP6 acupuncture group	23	54.26 ± 2.312	31.96 ± 3.082	0.000
GB39 control group	26	55.42 ± 1.842	36.58 ± 3.358	0.000
Nonacupoint control group	11	55.09 ± 3.755	30.00 ± 4.143	0.000
^□^P		0.928	0.419	

*P values are for within-group comparisons of the three groups.

^□^P values are for between-group comparisons of the three groups. VAS indicates visual analogue scale.

### Ratings of *Deqi* sensations

Type of needling sensations was recorded. And the proportion of patients with particular sensation was shown in Table 2. It should be noted that these data reflected the fact that the majority of patients recorded more than one sensation. In Table 3, there were no significant differences among the intensity of seven sensations.

**Table 2:** Frequency of deqi sensations in the three groups

**Sensations**	**SP6 acupuncture group**	**GB39 control group**	**Nonacupoint control group**	**P**
	**(N = 23)**	**(N = 26)**	**(N = 11)**	
Soreness	11 (47.83%)	16 (61.54%)	5 (45.45%)	0.533
Numbness	12 (52.17%)	11 (42.31%)	6 (54.55%)	0.710
Heaviness	15 (65.22%)	20 (76.92%)	8 (72.73%)	0.660
Warmth	8 (34.78%)	7 (26.92%)	1 (0.90%)	0.285
Cold	7 (30.43%)	4 (15.38%)	0 (0%)	0.088
Dull pain	15 (65.22%)	17 (65.38%)	5 (45.45%)	0.473
Sharp pain	15 (65.22%)	21 (80.77%)	9 (81.82%)	0.385

**Table 3:** Comparisons of the intensity of deqi sensations among the three groups (mm, Mean ± SE)

**Sensations**	**SP6 acupuncture group**	**GB39 control group**	**Nonacupoint control group**	**P**
	**(N = 23)**	**(N = 26)**	**(N = 11)**	
Soreness	9.96 ± 2.95	12.46 ± 3.08	8.64 ± 3.82	0.701
Numbness	14.87 ± 4.35	8.04 ± 2.95	10.00 ± 4.52	0.392
Heaviness	24.57 ± 5.29	21.15 ± 3.66	22.73 ± 5.85	0.869
Warmth	7.17 ± 2.77	5.38 ± 2.25	4.55 ± 4.55	0.824
Cold	6.96 ± 3.40	1.50 ± 0.77	0.00 ± 0.00	0.105
Dull pain	19.13 ± 4.11	15.12 ± 3.18	12.27 ± 5.93	0.554
Sharp pain	27.72 ± 4.91	23.46 ± 4.51	25.00 ± 7.69	0.822

**Figure 1:**
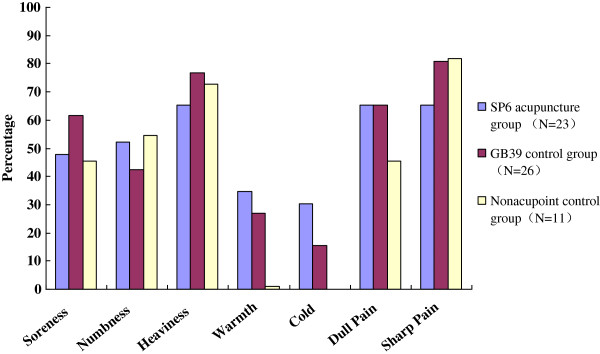
**Prevalence of various needling sensations in the three groups.** 100% indicates that the individual sensation occurs in all subjects.

Every sensation demonstrated some virtual differences in frequency of experience among three groups. Many sensations were shared by the *deqi* response generated at SP6, GB39 and nonacupoint. But more careful examination of the data revealed differences in frequency and intensity of individual sensations. Sharp pain, heaviness and dull pain were the most frequent sensation in SP6 acupuncture group and GB39 control group. However, followed by sharp pain and heaviness, numbness is another sensation occurred in the nonacupoint control group (Figure 1).

**Figure 2:**
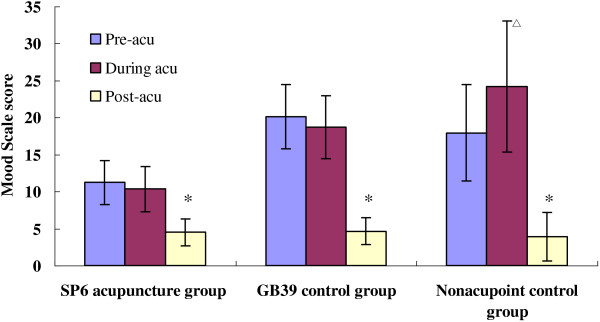
**Comparison of anxiety among three groups. **^△^ Compared with pre-acu P<0.05. ^*^ Compared with pre-acu P<0.05.

In Figure 2, it showed that patients receiving acupuncture at SP6 tended to relieve the pain intensity easier than those receiving acupuncture at GB39 or nonacupoint. In addition, most *deqi sensations* expensed to the “upper legs” and “lower legs” along the lower limbs.

## Discussion

This secondary analysis of data aimed to assess acupuncture needling sensations and the therapeutic effect of acupuncture. The results showed that pain relief occurred after acupuncture treatment in primary dysmenorrhea patients. But there exist no significant differences among three groups in terms of intensity of *deqi* feeling.

*Deqi* is a central concept in traditional Chinese acupuncture [2,15]. Many investigators have attempted to assess the relationship between *deqi* and therapeutic effects [16,17]. Some found better pain relief for acupuncture with *deqi*[18,19], whereas others did not [20,21]. This result was similar to the results of White P *et al.*, who suggested that the presence and intensity of *deqi* had no effect on pain relief for osteoarthritis (OA) patients [17].

*Deqi* is comprised of the sensation of the patient and the sensation of the acupuncturist [22]. Patients experienced *deqi* as multiple unique sensations at the needle site and surrounding regions [23]. Meanwhile, the perception of acupuncturist has been described as a slight pull of the needle downwards into the tissue [4,24,25]. It was recommended that the study should have frequent recording of *deqi*, using a much more sensitive measure and also be prudent to record any *deqi* noted by acupuncturists.

*Deqi* is difficult to study because of its subjective nature and multifactor influence. Factors such as patient’s body constitution, severity of the illness, acupoint location, needling techniques, manipulation skills of the acupuncturist, competence and understanding of the TCM theory, also play an important role in the therapeutic outcome [6].

Although this is not supported by current study that *deqi* is stronger at acupoints than at nonacupoints [26], sharp pain, heaviness and dull pain are the most frequent sensations in SP6 acupuncture group and GB39 control group. However, it was noted that numbness sensation occurred more often during acupuncture at nonacupoint, whereas the frequency of sharp pain and heaviness was similar to acupoints. Heaviness, aching, soreness, warmness and dull pain are conveyed by the slower-conducting Ad and C fibers, whereas numbness is conveyed by the faster-conducting Ab fibers [27]. Acupuncture at SP6 tended to relieve the pain intensity easier in primary dysmenorrhea patients than those receiving acupuncture at GB39 or nonacupoint.

An appropriate method of measuring *deqi* needs to be developed to support further acupuncture investigation. Researchers have sought to establish a credible rating scale for *deqi*, such as the Subjective Acupuncture Sensation Scale (SASS) [28,29], the MASS [13], the Southampton Needle Sensation Questionnaire [16] and the “*deqi* composite” [30]. In past decades, functional magnetic resonance imaging (fMRI) had been used to qualitatively and quantitatively characterize *deqi sensations*[31]. An fMRI study found strong *deqi sensations* induced strong deactivation of the limbic system [32]. Hui *et al.* found *deqi* response of acupoint stimulation likely arises from A-delta and C-fiber stimulation by the needle [30].

There are several limitations in this study. The decision of an appropriate control procedure for clinical studies on acupuncture is a particular challenge [7]. Previous acupuncture RCTs suggested that needling of acupoints was as effective as nonacupoints, in particular for pain relief, although both interventions were more effective than a waiting list control [21,33,34]. Few guidelines exist, however, for identifying appropriate sham point locations; the depth, direction, and duration of needle insertion; or the need for needle stimulation. Inserting a needle into any location is likely to have a physiological effect through a variety of mechanisms. It is still unknown what the sphere of influence is for local acupoints. For example, the distance of a point away from the needle-inserted acupoint that will not be also stimulated. Electrical stimulation was added after the initial *deqi sensations* was elicited for dysmenorrhea pain relief. Electro-acupuncture tends to elicit a strong “tingling” sensation which can easily mask pure *deqi sensations*. Besides, this is a secondary analysis of data from a RCT, which has a relatively big sample size and a statistical analysis in detail. However, in this paper, sample size is relatively small.

A power calculation was presented based on post hoc power analysis. This secondary data analysis was with a power of 25.44%. So this study may be underpowered to detect any differences. Possible reasons include that *deqi* is difficult to study because of its subjective nature and multifactor influence. Factors such as patient’s body constitution, severity of the illness, acupoint location, needling techniques, manipulation skills of the acupuncturist, competence and understanding of TCM theory, play important roles in the therapeutic outcome. Beside, patients experience *deqi* as multiple unique sensations at the needle site and surrounding regions. Multiple sensations usually present at the same time. It is underpowered to detect any differences for one single *deqi sensations*.

Due to the short term therapy and subjectivity of patients when rating the VAS scales, we found that the intensity of *deqi* during treatment does not relate to treatment outcome for primary dysmenorrhea pain. There are slight, but not clinical, differences in the most frequent sensations between acupoints and nonacupoints.

## Conclusions

There were no statistical differences among three groups in terms of intensity of *deqi* feeling. The types of sensation were similar across the groups with only minor differences among them. There existed no enough evidence for the role of *deqi* in acupuncture treatment. An appropriate method of measuring *deqi* needs to be developed to support further acupuncture investigation.

## Competing interests

The authors declare that they have no competing interests.

## Authors’ contributions

G-XS wrote and revised the manuscript, C-ZL and L-PW developed the original concepts for the review, Q-QLi, L-PG and M-MW have made substantial contributions to acquisition of data, analysis and interpretation of data; L-LH wrote the first draft of the paper. JW revised the manuscript. All authors contributed to the paper during development and read and approved the final version of the manuscript.

## Pre-publication history

The pre-publication history for this paper can be accessed here:

http://www.biomedcentral.com/1472-6882/14/69/prepub
